# Low back pain as the presenting sign in a patient with primary extradural melanoma of the thoracic spine - A metastatic disease 17 Years after complete surgical resection

**DOI:** 10.1186/1477-7819-9-150

**Published:** 2011-11-17

**Authors:** Darko Katalinic, Branimir Anic, Ranka Stern-Padovan, Miroslav Mayer, Mirna Sentic, Nada Cikes, Kamelija Zarkovic, Snjezana Dotlic, Stjepko Plestina

**Affiliations:** 1 Department of Oncology, University Hospital Centre (KBC Zagreb), University of Zagreb School of Medicine, Zagreb, Croatia; 2 Department of Clinical Immunology and Rheumatology, University Hospital Centre (KBC Zagreb), University of Zagreb School of Medicine, Zagreb, Croatia; 3 Department of Diagnostic and Interventional Radiology, University Hospital Centre (KBC Zagreb), University of Zagreb School of Medicine, Zagreb, Croatia; 4 Department of Pathology and Cytology, University Hospital Centre (KBC Zagreb), University of Zagreb School of Medicine, Zagreb, Croatia

**Keywords:** primary spinal melanoma, metastatic disease, low back pain

## Abstract

Primary spinal melanomas are extremely rare lesions. In 1906, Hirschberg reported the first primary spinal melanoma, and since then only 40 new cases have been reported. A 47-year-old man was admitted suffering from low back pain, fatigue and loss of body weight persisting for three months. He had a 17-year-old history of an operated primary spinal melanoma from T7-T9, which had remained stable for these 17 years. Routine laboratory findings and clinical symptoms aroused suspicion of a metastatic disease. Multislice computed tomography and magnetic resonance imaging revealed stage-IV melanoma with thoracic, abdominal and skeletal metastases without the recurrence of the primary process. Transiliac crest core bone biopsy confirmed the diagnosis of metastatic melanoma. It is important to know that in all cases of back ore skeletal pain and unexplained weight loss, malignancy must always be considered in the differential diagnosis, especially in the subjects with a positive medical history. Patients who have back, skeletal, or joint pain that is unresponsive to a few weeks of conservative treatment or have known risk factors with or without serious etiology, are candidates for imaging studies. The present case demonstrates that complete surgical resection alone may result in a favourable outcome, but regular medical follow-up for an extended period, with the purpose of an early detection of a metastatic disease, is highly recommended.

## Background

Malignant melanoma is an aggressive tumour with a variable prognosis: that depends on tumour site and the extent of surgical resection. According to epidemiological data, its annual incidence has increased significantly in recent years [1]. Primary melanoma of the central nervous system (CNS) is rare and accounts for approximately 1% of all cases of melanoma [2]. They are either intradural, extradural or have both intra and extradural components. However, primary spinal extradural melanoma is extremely rare [3]. It is a solitary lesion associated with leptomeninges and neural crest, the tissue source of its precursory melanocytes. In 1906, Hirschberg reported the first primary spinal melanoma, and since then only 40 new cases have been reported [4]. We present a case of primary thoracic extradural spinal melanoma occurring with low back pain and metastatic disease 17 years after the initial surgical resection.

## Case presentation

**Figure 1:**
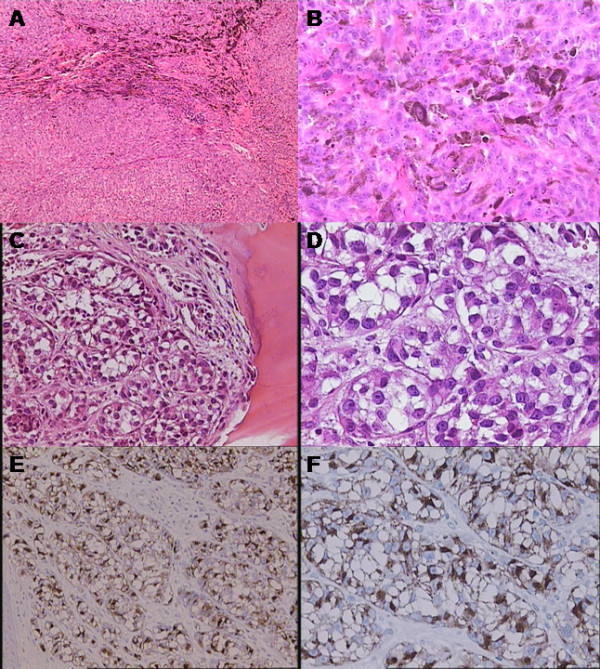
**Histopathological evaluation**. Hematoxylin and eosin histologic analysis revealed a highly cellular malignant tumour with prominent nucleoli (image A 100×, images B and C 200×, image D 400×). Immunohistochemical study shows that the tumour cells stained diffusely positive for Melan-A and HMB-45, consistent with the diagnosis of melanoma (images E 200×, image F 400×).

In 1993, a 30-year-old man without accompanying comorbidity, was hospitalized for a sudden bilateral spastic paraparesis. General physical examination revealed progressive bilateral lower extremity weakness associated with hypoesthesia distal of the dermatome level of T9. A plain myelography showed epidural block of contrast flowing along the spinal canal from the level of T7. Therefore, laminectomy was performed at the T7-T9 level which revealed a dark, pigmented, extradurally located tumour measuring 2.5 × 1 cm., without infiltration of dura and spreading through vertebral foramina. Histopathologic and immunohistochemical analysis confirmed a malignant melanoma (Figure 1A and 1B). The patient did not receive any radiotherapy because surgical resection appeared completely without opening the dura. The patient was also subjected to further clinical and oncological evaluation which did not reveal any other melanoma lesions or metastases and other primary process could not be established. With these data and according to Hayward's criteria [5], the diagnosis of primary spinal melanoma was established. He was discharged from the hospital in a good general condition with the recommendation for a regular follow-up and he was cleared disease-free a few years after the operation without any need for specific oncological treatment.

**Figure 2:**
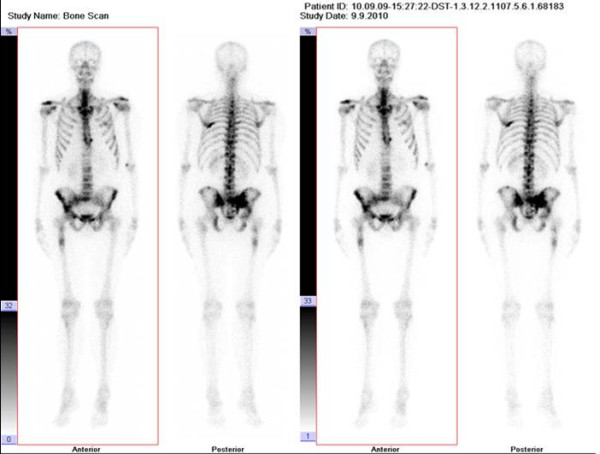
**Bone scintigraphy**. Bone scintigraphy with Tc-99 m demonstrates extensive osteoblastic activity, predominantly located in the axial skeleton within the spine, pelvis and ribs.

**Figure 3:**
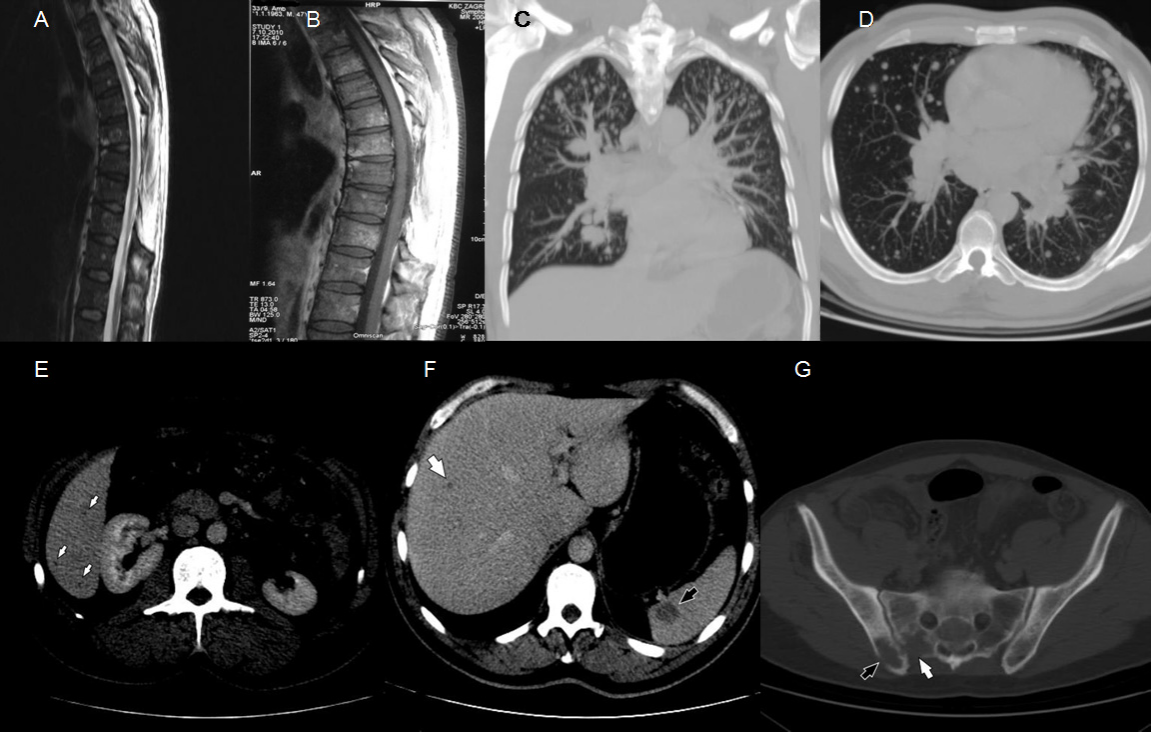
**Radiological evaluation**. Magnetic resonance sagittal T2 weighted image (image A) and postcontrast sagittal T1 image (image B) of the thoracic spine shows millimetric hyperintense metastases with no residual-recurrent tumor. Multislice computed tomography of the chest and abdomen demonstrated osteolytic (image G) and parenchymal metastases (arrows) in lungs (images C and D), liver and spleen (images E and F).

In 2010, 17 years later, the same patient was admitted to our rheumatology department with a three-month history of low back pain, fatigue and loss of body weight. He was treated with medication and physiotherapy, but his symptoms did not subside. Physical examination showed a right sacroiliac joint tenderness on palpation with a positive Mennel's sign, Lasègue's sign was bilaterally negative and the lumbar vertebral segment motility was very painful. In this period he had no history of fever or pain in other joints, bones and muscles. He did not notice any skin lesions and he didn't have any other problems other than the above. The stool and urine were normal. General physical examination revealed no signs of lymphadenopathy, splenomegaly, hepatomegaly or other abnormalities. Routine laboratory tests showed anemia (hemoglobin 102 g/L, range 138-175), thrombocytosis (641 × 10^9^/L, range 158-424), low serum iron concentration (3 μmol/L, range 8-30), high serum ferritin concentration (790 μg/L, range 15-150), high serum lactate-dehydrogenase activity (745 U/L, range 1-241) and high erythrocyte sedimentation rate (96 mm/h, range 3-23). Further laboratory studies and blood chemistry including serum glucose level, alkaline phosphatase, renal and hepatic parameters, electrolytes, protein electrophoresis, coagulogram, tumour markers, urinanlysis, were all within normal limits. We suspected he had developed a metastatic disease because the patient had a history of primary spinal melanoma. Chest radiography showed a right hilar lymphadenopathy and pulmonary metastatic lesions. Pelvis and thoracolumbosacral plain radiographs revealed multiple metastatic lesions. Tc-99 m bone scintigraphy showed increased osteoblastic activity, which predominantly affected the axial skeleton. (Figure 2). Transiliac crest core bone biopsy showed tumour tissue composed of large melanocytes with prominent nucleoli, which makes up 90% of all bone marrow cells (Figure 1C and 1D). Immunohystochemically, tumour cells give a positive reaction to Melan-A and HMB-45 consistent with the diagnosis of metastatic melanoma (Figure 1E and 1F). A staging contrast enhanced multislice computed tomography (MSCT) of the chest, abdomen and pelvis revealed stage-IV melanoma with thoracic lymphadenopathy, multiple osteolytic lesions and numerous metastases within lung parenchyma, liver and spleen (Figure 3C-3G). The control magnetic resonance imaging (MRI) of the thoracic spine showed no recurrence of the primary process (Figure 3A and 3B). The patient was seen by a medical oncologist with a view to chemotherapy. We have decided to start with Dartmouth regimen (dacarbazine, cisplatin, carmustine, and tamoxifen) which has been reported to induce major tumour responses in 40% to 50% of stage-IV melanoma patients [6]. He has received only three cycles of Dartmouth regimen and he tolerated the treatment very well. Unfortunately, during his next hospitalization he died as a result of febrile neutropenia, sepsis and multiple organ failure.

## Discussion

Melanomas could occur in any tissue in which melanocytes can normally be found. Primary spinal melanomas are exceedingly rare malignant tumours whose origin can be illustrated by embriology. In 1859, Virchow showed that primary melanomas of the CNS developed from melanocytes located in the leptomeninges. Melanoblasts, the precursors of melanocytes are belived to be derived from the neural crest during early embrionic development. The presence of melanocytes in neural tissue can be explained by the fact that these melanocytes accompany blood vessels to the leptomeninges and can proliferate in the neural parenchyma [4,7]. They can also be precursors to benign melanocytoma, pigmented schwannoma and pigmented medulloblastoma, which is relevant for differential diagnosis [4,8]. A big difficulty when discussing melanoma of the CNS is to decide which cases represent primary growths and which are metastatic disease. In general, melanocytic mass in the CNS is known to be the result of metastasis. Only after detailed clinical work-up including dermatological, ophtalmological and endoscopic examination, a solitary melanotic lesion of the CNS can be identified as a primary tumour. The following criteria should also be considered: no melanoma tumour outside the CNS and involvement of the meninges and/or neural tissue [5]. In all cases of primary spinal melanoma, the clinical presentation suggests progressive spinal cord compression with clinical signs and symptoms of compressive myelopathy. The neurosurgical treatment is the method of choice, but in some cases where surgery is not possible, radiotherapy also may be used. Larson et al. [9] showed an average life expectancy of approximately seven years after surgery with radiotherapy. As opposed to that, the survival time with melanoma of the skin with metastases to CNS was only six months [9]. Considering the well-known metastazing potential of melanomas, it may seem surprising that primary CNS melanomas do not spread for a long time and may have a favourable outcome after complete neurosurgical excision. Clinically, this is the main reason why distinction between primary and metastatic melanoma is important. In the present case, progression of melanoma was controlled for 17 years, which is an unusually long time. The key factor in this successful, long survival remains unclear. The characteristics of the CNS probably contribute to the nature of this disease and contrary to melanoma of the skin, lymphatic spread would be impossible because there are no lymphatic vessels in the neural tissue. The blood-brain barrier of the neural parenchyma probably also prevents the hematogenous spreading.

At present, MRI has a leading diagnostic role in patients with serious spinal pathology but distinction of tumour type based on MRI findings remains difficult. The MRI findings of melanoma vary deeply depending on the amount of melanin-containing cells and on the presence or absence of hemorrhage [10,11]. The paramagnetic effect that derives from the presence of organic radicals from melanin is responsible for hyperintensity on T1 weighted images and hypointensity on T2 weighted images. Although MRI is the primary imaging modality in diagnosis of spinal tumors, histopathologic diagnosis is required to differentiate benign pathologies from malignant disease. Bony metastases of melanoma exhibit a predilection to spread in the axial skeleton with an incidence of 70-86% [12]. Plain radiography findings of bony metastasis of melanoma are ostelytic lesions, which can not be distinguished from other osteolytic metastases on the basis of imaging criteria. Conventional plain radiographs have been reported to identify approximately 40% of metastatic bone lesions [13]. Radionuclide imaging of bone marrow metastases with a Tc-99 m is very effective, simple and safe method for the detection of melanoma metastases or recurrent disease [12]. Bone scans remain the study of choice for initial screening, because of their overall sensitivity, lower cost and ability to assess the entire body conveniently [12]. Since computed tomography (CT) facilitates simultaneous examination of multiple sites at risk for metastatic disease and may allow earlier detection of metastatic disease, CT is being used with an increasing frequency for clinical staging of melanoma. It is also being used to monitor clinical response of patients to different therapy [14]. Whole body positron-emission tomography-CT (PET-CT) was found to be capable of uncovering metastases not seen with conventional CT alone and could also be used for initial staging [15]. Thus, the ultimate diagnosis can only be made on the basis of histopathological and immunohystochemical examination.

Melanoma is considered a relatively radioresistant tumour [16]. However, some studies have shown that patients with spinal cord compression may achieve shrinkage of the tumor with radiation therapy [17,18]. Radiotherapy is reserved for palliation of symptoms due to local tumour growth [16], as sole decompressive modality or as an adjuvant to laminectomy [19,20]. Irradiation regimens should be selected on the basis of patient long-term or short-term prognosis, and on spinal cord tolerance which limits the utilisation of high dose per fraction schedules (30 Gy at 2-3 Gy per day in 15-20 days). In 1995, Hamilton and colleagues [21] first described the use of linear-accelerator-based spinal stereotactic radiosurgery. Most radiosurgical departments currently utilize doses of 12-24 Gy to the margin of the treatment volume and deliver spinal radiosurgery in 1-5 fractions [22,23]. The prescription dose depends in part upon the tumor location and volume as well as the fractionation scheme, surrounding organs at risk, and prior radiotherapy.

Melanoma has also been refractory to most standard systemic therapy for decades. Single-agent or combination chemotherapy drugs in use at this time are of limited value. The two approved treatments, dacarbazine and interleukin-2, have not demonstrated an impact on overall survival. Some novel regimens (multiagent chemotherapy, biochemotherapy, biotherapy with interferon-γ and interferon-α, antisense bcl-2 oligonucleotide oblimersen, a reactive-oxide species inhibitor or a combined approach) [20-31] have also not significantly impacted overall survival [32]. However, evidence of tumor regression with prolonged time to progression has been seen in patients with melanoma who received cytotoxic T lymphocyte antibodies (CTLA-4) [33]. Combination of chemotherapy and/or new monoclonal antibody that overcomes CTLA-4-mediated T-cell suppression (ipilimumab) improved overall survival [34,35]. Phase 1 and 2 clinical trials of the BRAF kinase inhibitor vemurafenib produced improved rates of overall and progression-free survival in patients with previously untreated melanoma with the BRAF V600E mutation [36]. Our understanding of the biology of melanoma is steadily increasing and there will be a number of new agents in the future which have already entered clinical trials and are promising combined approaches targeting melanoma by different molecular mechanisms.

Finally, early detection of melanoma through patient education regarding clinical characteristics of melanoma, counselling on the risk of developing a metastatic tumor, self-examination and medical examination continues to be of the utmost importance. During melanoma follow-up, patients are clinically monitored in order to detect a relapse. There is currently no scientific consensus on the frequency of follow-up after initial surgical treatment and the use of imaging techniques. Typically patients will be seen every 3-6 months during the first 3 years and every 6-12 months thereafter. Routine laboratory tests, tumor markers (S 100B, tyronsinase), ultrasound of lymph nodes, chest radiography, CT or whole body PET/PET-CT scans may be used in the follow-up of patients with primary melanoma or following treatment of metastases [37,38].

## Conclusion

This case remind us how little we truly know about the biological behavior of melanoma and the patient's inherent biological response to the tumor. It is important to know that in all cases of back ore skeletal pain and unexplained weight loss, malignancy must always be considered in the differential diagnosis, especially in the subjects with a positive medical history. Patients who have back, skeletal, or joint pain that is unresponsive to a few weeks of conservative treatment or have known risk factors with or without serious etiology, are candidates for imaging studies.

Melanomas are unpredictable tumours with a different clinical course. The present case also demonstrates that complete resection alone may result in a favourable outcome, but regular medical follow-up for an extended period, patient education and councelling with the purpose of an early detection of a metastatic disease, is highly recommended.

## Consent

Written informed consent was obtained from the patient for publication of this Case report and any accompanying images. A copy of the written consent is available for review by the Editor-in-Chief of this journal.

## Abbreviations

CNS: central nervous system; MSCT: multislice computed tomography; MRI: magnetic resonance imaging (MRI); CT: computed tomography; PET-CT: positron-emission tomography; Gy: gray; CTLA-4: cytotoxic T lymphocyte antibodies; BRAF: proto-oncogene BRAF

## Competing interests

Each coauthor certifies that he ore she has no commercial association that might pose a conflict of interest in connection with the submitted article.

## Authors' contributions

DK, BA and MM carried out the study design and writing. MS and NC participated in the data collection and interpretation and drafted the manuscript. RSP, KZ and SD participated in the design of the study and the figure design. SP participated in the literature search and helped to draft the manuscript. All authors read and approved the final manuscript.
